# Effect of vitamin B supplementation on cancer incidence, death due to cancer, and total mortality: A PRISMA-compliant cumulative meta-analysis of randomized controlled trials

**DOI:** 10.1097/MD.0000000000003485

**Published:** 2016-08-07

**Authors:** Sui-Liang Zhang, Ting-Song Chen, Chen-Yun Ma, Yong-Bin Meng, Yu-Fei Zhang, Yi-Wei Chen, Yu-Hao Zhou

**Affiliations:** a Department of Invasive Technology, Shanghai Seventh People's Hospital; b Department of Traditional Chinese and Western Medicine, Eastern Hepatobiliary Surgery Hospital, Second Military Medical University; c Department of Medical Laboratory, Shanghai Seventh People's Hospital; d Department of Traditional Chinese Medicine, Changhai Hospital, Second Military Medical University; e Department of Rehabilitation Institute, Shanghai Seventh People's Hospital, Shanghai, China.

**Keywords:** cancer, meta-analysis, mortality, vitamin B

## Abstract

**Background:**

Observational studies have suggested that vitamin B supplementation is associated with cancer risk, but this association remains controversial. A pooled data-based meta-analysis was conducted to summarize the evidence from randomized controlled trials (RCTs) investigating the effects of vitamin B supplementation on cancer incidence, death due to cancer, and total mortality.

**Methods:**

PubMed, EmBase, and the Cochrane Library databases were searched to identify trials to fit our analysis through August 2015. Relative risk (RR) was used to measure the effect of vitamin B supplementation on the risk of cancer incidence, death due to cancer, and total mortality using a random-effect model. Cumulative meta-analysis, sensitivity analysis, subgroup analysis, heterogeneity tests, and tests for publication bias were also conducted.

**Results:**

Eighteen RCTs reporting the data on 74,498 individuals were included in the meta-analysis. Sixteen of these trials included 4103 cases of cancer; in 6 trials, 731 cancer-related deaths occurred; and in 15 trials, 7046 deaths occurred. Vitamin B supplementation had little or no effect on the incidence of cancer (RR: 1.04; 95% confidence interval [CI]: 0.98–1.10; *P* = 0.216), death due to cancer (RR, 1.05; 95% CI: 0.90–1.22; *P* = 0.521), and total mortality (RR, 1.00; 95% CI: 0.94–1.06; *P* = 0.952). Upon performing a cumulative meta-analysis for cancer incidence, death due to cancer, and total mortality, the nonsignificance of the effect of vitamin B persisted. With respect to specific types of cancer, vitamin B supplementation significantly reduced the risk of skin melanoma (RR, 0.47; 95% CI: 0.23–0.94; *P* = 0.032).

**Conclusion:**

Vitamin B supplementation does not have an effect on cancer incidence, death due to cancer, or total mortality. It is associated with a lower risk of skin melanoma, but has no effect on other cancers.

## 1 Introduction

The potential role of vitamin B in relation to the risk of cancer, including breast and colorectal cancer, has been investigated in several observational studies.^[1–3]^ Although the mechanism of action is unclear, vitamin B may affect the incidence of cancer because it is essential for the biosynthesis of nucleotides, replication of DNA, supply of methyl-groups, and the growth and repair of cells.^[4–7]^ However, observational studies often overestimate the magnitude of the effect and do not prove causality, and the effect of vitamin B supplementation on the risk of cancer has not been confirmed by randomized controlled trials (RCTs).^[8–25]^ Finally, previous studies have not investigated the potential interaction of supplementation with both vitamin B6 and B12 and its effect on cancer risk.^[26,27]^

The reasons for the discordance between the findings of RCTs^[8–25]^ and earlier observational studies^[1–3]^ could be as follows: individual trials might have been underpowered to show clinical benefit, especially if event rates were lower than expected, which always acquired broad confidence intervals; the duration of follow-up was shorter than that needed to show a clinical benefit, or different types of supplements might provide a biased view of the study question; observational studies are hypothesis-generating but cannot prove causality, and always overestimate the magnitude of the effect; and most trials were designed with vascular events as the primary endpoint, and their sample size did not allow adequate power to detect potential clinically relevant differences in cancer incidence.

The effect of vitamin B supplementation on primary and secondary prevention of adverse cardiovascular outcomes has been studied in numerous RCTs.^[8–25]^ With long-term follow-up and collection of cancer data in a majority of studies, insight into the risk of cancer among participants with vitamin B supplementation and those with placebo can be derived. In this study, a meta-analysis of RCTs was conducted to evaluate the effect of vitamin B on cancer incidence, death due to cancer, and total mortality in specific subpopulations, in an attempt to determine the effect of folic supplementation interaction with vitamin B6 and B12 on the risk of cancer-related outcomes. In addition, cumulative meta-analyses were employed to determine the evidence base for routine vitamin B supplementation in clinical practice.

## 2 Methods

### 2.1 Data sources, search strategy, and selection criteria

This review and pooled data based meta-analysis was conducted and reported according to the Preferred Reporting Items for Systematic Reviews and Meta-Analysis (PRISMA) Statement, issued in 2009.^[28]^ Ethics approval was not necessary for this study, as only deidentified pooled data from individual studies were analyzed. RCTs on vitamin B supplementation, written in the English language, were eligible for inclusion in our study, regardless of the publication status (published, in press, or in progress), and the effects of vitamin B supplementation on cancer incidence, death due to cancer, total mortality, and any specific-type cancer were examined. We systematically searched the PubMed, EmBase, and Cochrane Central Register of Controlled Trials to identify all the trials related to vitamin B supplementation through August 2015. The electronic databases were searched using the following keywords.

“vitamin B” AND “randomized controlled trials” AND “clinical trials” AND “human” AND “English.” Furthermore, ongoing trials were identified from the metaRegister of Controlled Trials. Finally, manual searches were performed from the reference lists within the entire relevant nonrandomized controlled trials in order to identify the additional eligible studies.

According to a standardized approach, 2 authors carried out literature search, data extraction, and quality assessment independently. The primary author solved any differences until a consensus was achieved if there were any inconsistencies between 2 authors. In order to avoid less confounding variables or biases, we restricted our study design to RCTs rather than observational studies. A study was eligible for inclusion if the following criteria were met: it was an RCT; the trial evaluated the effects of vitamin B supplementation compared with those of placebo or low-dose vitamin B; a follow-up period was of at least 1 year; and the trial reported at least 1 outcome as either cancer incidence or death due to cancer.

### 2.2 Data collection and quality assessment

A standard protocol was adopted independently by 2 authors to extract the data from all included trials, and any differentials between these 2 authors were resolved for an agreement though a group discussion. The collected data include study characteristics (first author or study group's name, publication year, study design, type of blinding, intervention regimes, controls, and the duration of follow-up.), participants’ characteristics (number of patients, mean age, percentage of men, background fortification, current diseases status, baseline total homocysteine level, and baseline folate level), and outcomes variables (cancer incidence, death due to cancer, total mortality, and specific-type cancer incidence). Simultaneously, the quality of included trials was evaluated using Jadad score^[29]^ which ranged from 0 to 5, and based on the following items such as randomization, concealment of the treatment allocation, blinding, completeness of follow-up, and the use of intention-to-treat analysis. In our analysis, we considered a study with a score of 4 or 5 to be of high quality.

### 2.3 Statistical analysis

The results of each RCT were assigned as dichotomous frequency data, and the event numbers were extracted from each trial to calculate relative risks (RRs) and 95% confidence intervals (CIs) of each individual trial. The overall RRs with 95% CIs were calculated for cancer incidence, death due to cancer, total mortality, and specific-type cancer in participants who received vitamin B supplementation. The comparison of pooled RR between vitamin B supplementation and placebo was performed using fixed-effect and random-effects models respectively, and then the results from the random-effects model were presented here.^[30,31]^ The heterogeneity of the treatment effects among included trials was evaluated using Q statistic; meanwhile, a *P* value for heterogeneity of less than 0.10 was considered to be statistically significant.^[32,33]^ In the cumulative meta-analysis, outcome data for cancer incidence, death due to cancer, and total mortality were shown sequentially in light of the year included in trials which first became available.

Potential heterogeneity in estimates of the treatment effects was explored using univariate meta-regression.^[34]^ Subgroup analyses were also performed for cancer incidence. The estimates between 2 subsets were compared by using interaction tests, which were based on Student *t* distribution rather than on normal distribution as the number of included studies was small.^[35]^ Sensitivity analyses by removing each individual trial from the meta-analysis were also conducted.^[36]^ The publication bias for cancer incidence, death due to cancer, and total mortality was statistically assessed using funnel plots, Egger^[37]^ and Begg tests,^[38]^ and *P* values less than 0.05 was considered to be statistically significant. STATA software (Version 10.0; Stata Corporation, College Station, TX, USA) was used to perform the statistical analyses.

## 3 Results

**Figure 1:**
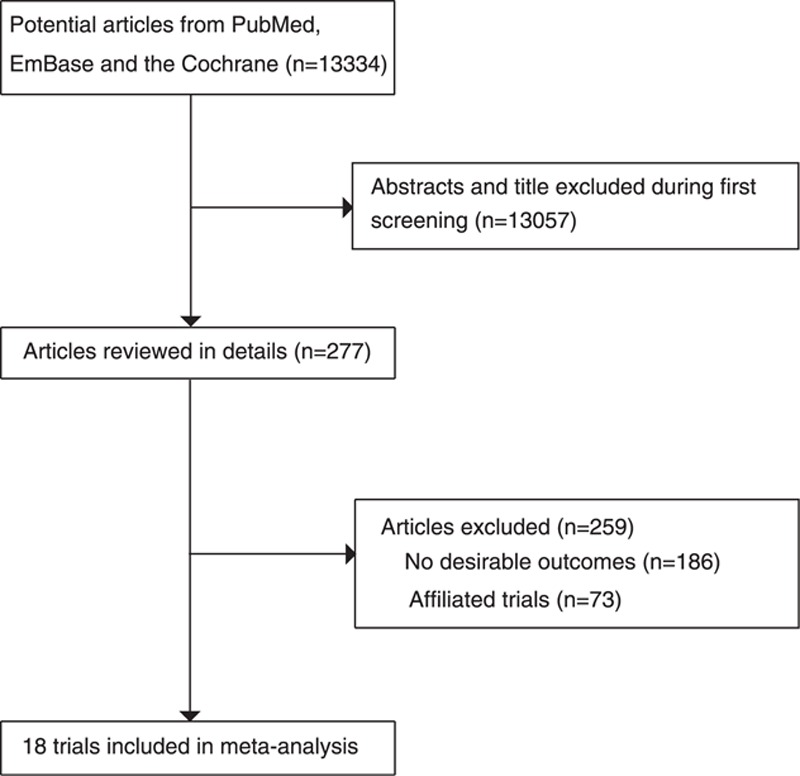
Flow diagram of the literature search and trials selection process.

The primary electronic search produced 13,334 articles in total. After scanning titles and abstracts, 13,057 irrelevant or duplicate articles were excluded during the initial review. The remaining 277 potentially eligible articles were retrieved after detailed evaluations. Finally, 18 RCTs^[8–25]^ were eligible for pooled analysis (Fig. 1). Table 1 presents the general characteristics of these included trials and baseline information of total 74,498 individuals. Of these, 3 trials^[8,12,14]^ evaluated vitamin B supplementation in patients with chronic renal disease or end-stage renal disease, 7 trials^[9–11,15,18–20]^ reported patients with cardiovascular disease, 3 trials^[13,21,23]^ evaluated patients with a recent history of colorectal adenomas and no previous invasive large intestine carcinoma, and the remaining 5 trials^[16,17,22,24,25]^ reported participants with cardiovascular risk factors. The number of cases in each included trial ranged from 114 to 20,702 during the follow-up time of 2.0 to 7.3 years, the baseline homocysteine level ranged from 9.6 to 31.7 μmol/L, the baseline folate level ranged from 8.1 to 35.34 nmol/L, and the net change in total homocysteine level ranged from −2.1 to −15.1 μmol/L. In the intervention groups, the dose of folic acid ranged from 0.4 to 40 mg per day, that of vitamin B6 from 3.0 to 250 mg per day, and that of vitamin B12 from 20 to 2000 μg per day. The breakdown for the number of trials available for each outcome was 16, 6, and 15 for cancer incidence,^[9–11,13–25]^ death due to cancer,^[6,8,10,12,16,20]^ and total mortality,^[8–11,13–21,23,24]^ respectively. The quality of the trials was assessed using the Jadad score.^[29]^ We considered a score ≥4 to indicate a high-quality study. According to the Jaded scoring method, 6 trials^[8,9,11,19,20,25]^ scored 5 points, another 6 trials^[10,13–15,18,24]^ scored 4 points, 3^[16,17,22]^ scored 3 points, 2^[12,21]^ scored 2 points, and the remaining 1^[23]^ scored 1 point.

**Table 1:**
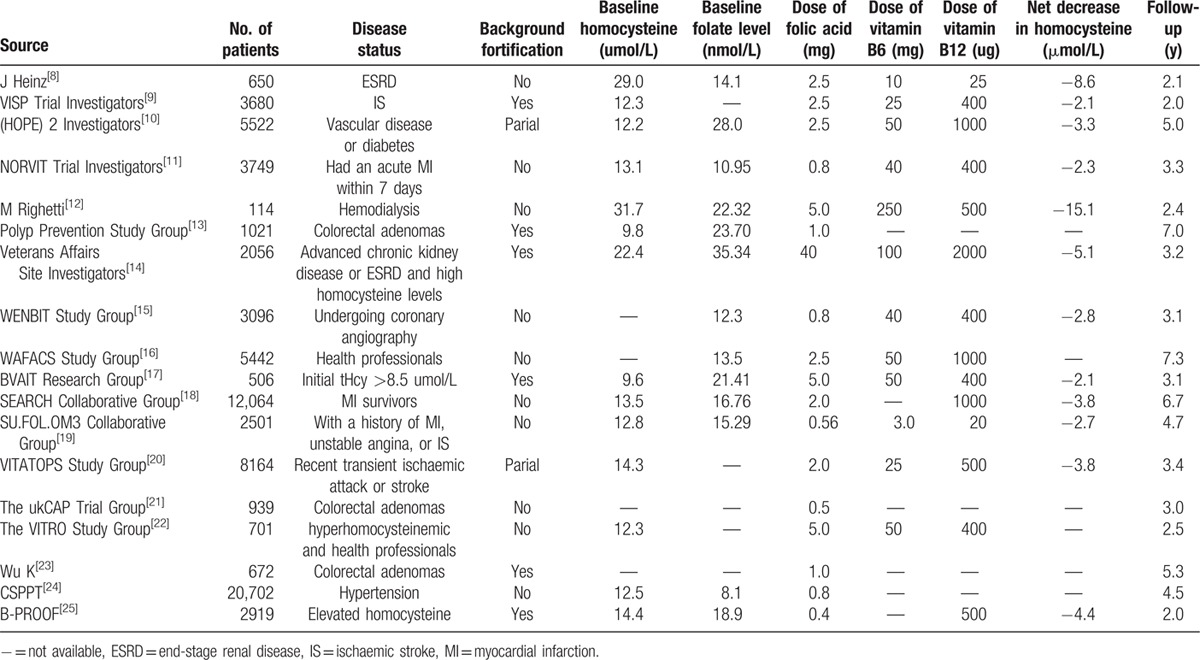
Design and characteristic of trials included in our meta-analysis.

**Figure 2:**
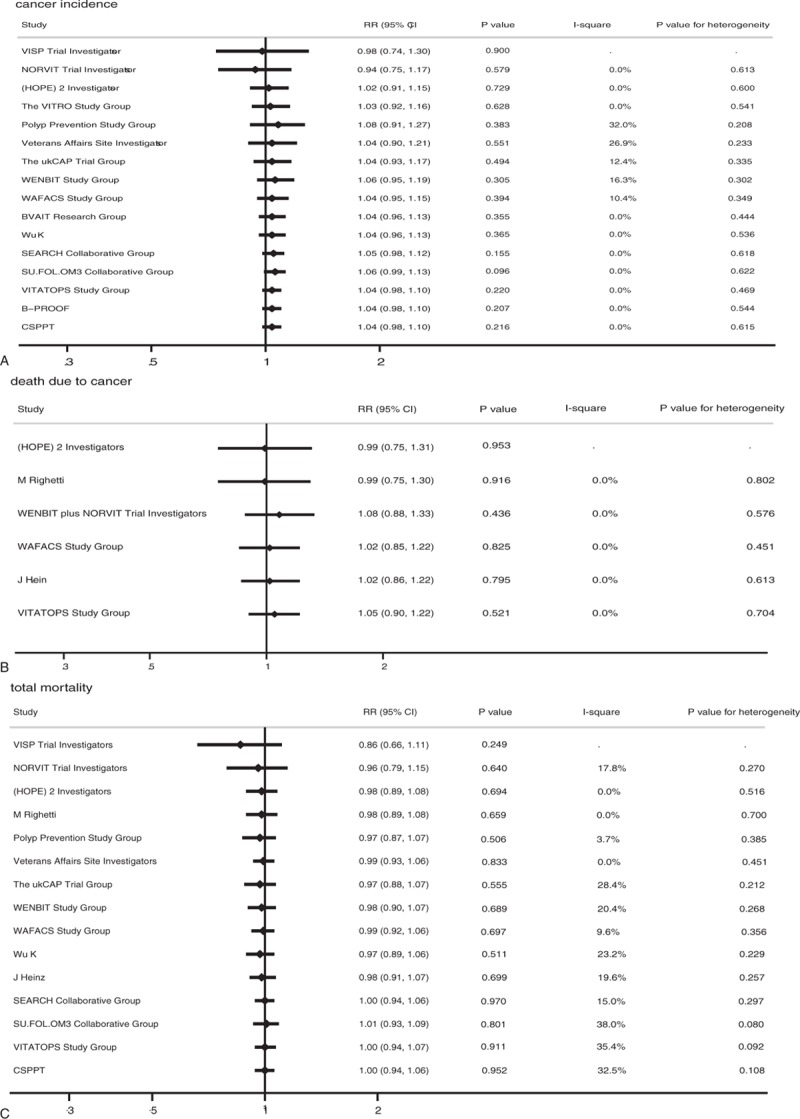
Cumulative meta-analysis of the effect of vitamin B supplementation on the risk of cancer incidence (A), death due to cancer (B), and total mortality (C).

Data from 73,269 participants were used to evaluate the effect of vitamin B supplementation on cancer incidence and included 4103 cancer events. Vitamin B supplementation caused an increase of 4% in cancer incidence; however, this was not a significant change (RR: 1.04; 95% CI: 0.98–1.10; *P* = 0.216; without evidence of heterogeneity; Fig. 2A).

Data from 26,729 participants were used to evaluate the effect of vitamin B supplementation on death due to cancer and included 731 cases of cancer-related mortality. Vitamin B supplementation increased the death rate due to cancer by 5%, but the change was not significant (RR, 1.05; 95% CI: 0.90–1.22; *P* = 0.521; without evidence of heterogeneity; Fig. 2B).

Data from 69,744 participants were used to evaluate the effect of vitamin B supplementation on total mortality and included 7046 death events. There were no significant differences in total mortality between participants receiving vitamin B and those receiving placebo (RR, 1.00; 95% CI: 0.94–1.06; *P* = 0.952; with moderate heterogeneity; Fig. 2C). A sensitivity analysis was conducted for total mortality. However, after sequential exclusion of each trial, the conclusion was not affected by the exclusion of any specific trial.

When a cumulative meta-analysis for cancer incidence was carried out, the original nonsignificant result for an effect of vitamin B persisted; the effect was slight and borderline nonsignificant. Similarly, the nonsignificant result persisted when cumulative meta-analyses for death due to cancer and total mortality were conducted.

**Figure 3:**
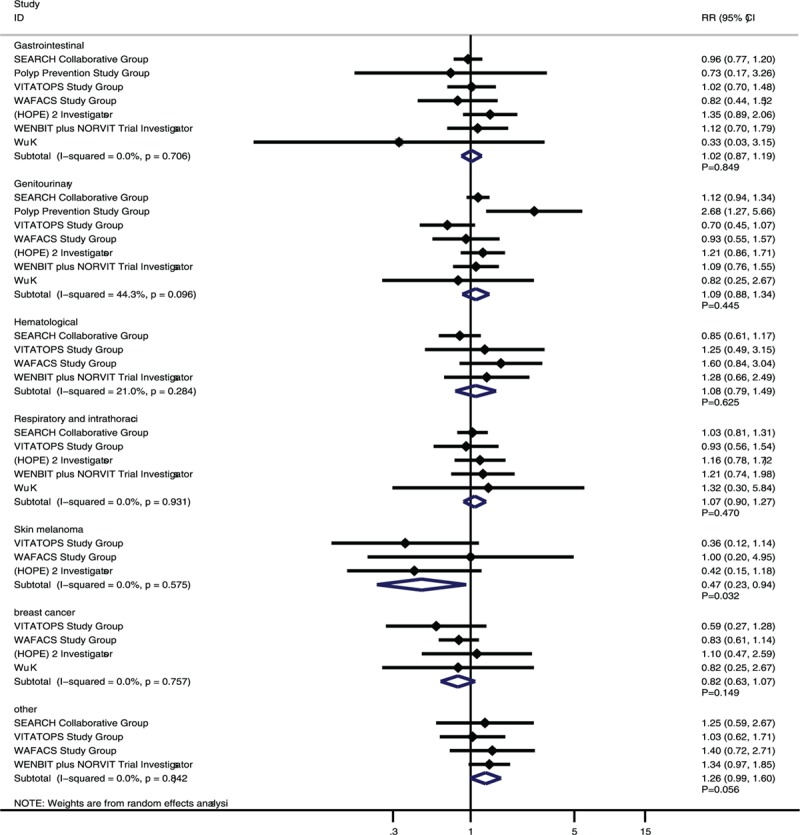
Effect of vitamin B supplementation on specific-type cancer.

The effects of vitamin B supplementation on the risk of specific types of cancer were also evaluated. Overall, vitamin B supplementation was associated with a significantly reduced risk of skin melanoma (RR, 0.47; 95% CI: 0.23–0.94; *P* = 0.032; Fig. 3), whereas it had no significant effect on the risk of gastrointestinal cancer (RR, 1.02; 95% CI: 0.87–1.19; *P* = 0.849), genitourinary cancer (RR, 1.09; 95% CI: 0.88–1.34; *P* = 0.445), hematological cancer (RR, 1.08; 95% CI: 0.79–1.49; *P* = 0.625), respiratory and intrathoracic cancer (RR, 1.07; 95% CI: 0.90–1.27; *P* = 0.470), breast cancer (RR, 0.82; 95% CI: 0.63–1.07; *P* = 0.149), and other types of cancers (RR, 1.26; 95% CI: 0.99–1.60; *P* = 0.056).

Heterogeneity testing for the analysis showed a *P* >0.10 for cancer incidence and death due to cancer; no significant heterogeneity was observed in the overall analysis, which suggests that most variation was attributable to chance alone. However, moderate heterogeneity was observed in the magnitude of the effect on total mortality across the trials. Meta-regression analyses were performed^[34]^ for cancer incidence that included the mean age, baseline homocysteine level, baseline folate level, dose of folic acid, dose of vitamin B6, dose of vitamin B12, and duration of follow-up. The results indicated that these variables were not significant factors contributing to the association between vitamin B supplementation and cancer incidence (data not shown).

**Figure 4:**
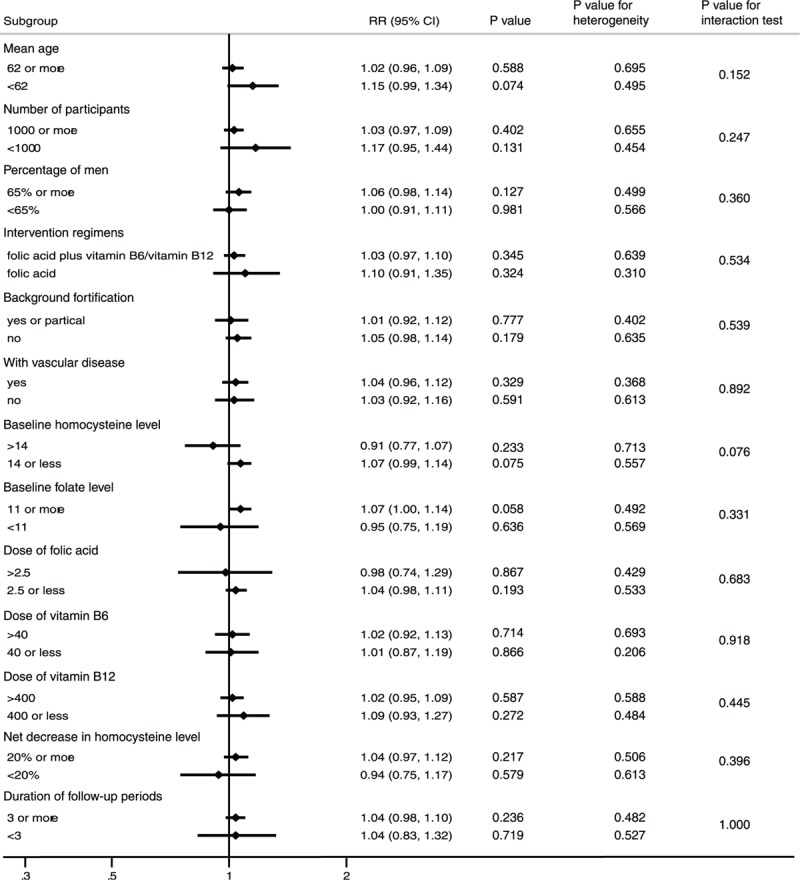
Subgroup analysis for cancer incidence.

Subgroup analyses were conducted for cancer incidence, death due to cancer, and total mortality to minimize heterogeneity and explore the effect of vitamin B supplementation in any specific subpopulations. Vitamin B supplementation might play an important role in cancer incidence if the mean age of the participants is <62 years (RR, 1.15; 95% CI: 0.99–1.34; *P* = 0.074; Fig. 4), and baseline homocysteine levels are <14 μmol/L (RR, 1.07; 95% CI: 0.99–1.14; *P* = 0.075; Fig. 4), although these results were not statistically significant. When subgroup analyses based on other factors were carried out, no significant differences were observed between vitamin B supplementation and placebo. Furthermore, there was no significant difference in the effect of vitamin B supplementation between the 2 subgroups with respect to cancer incidence. Finally, similar nonsignificant results were detected for death due to cancer and total mortality (data not shown).

**Figure 5:**
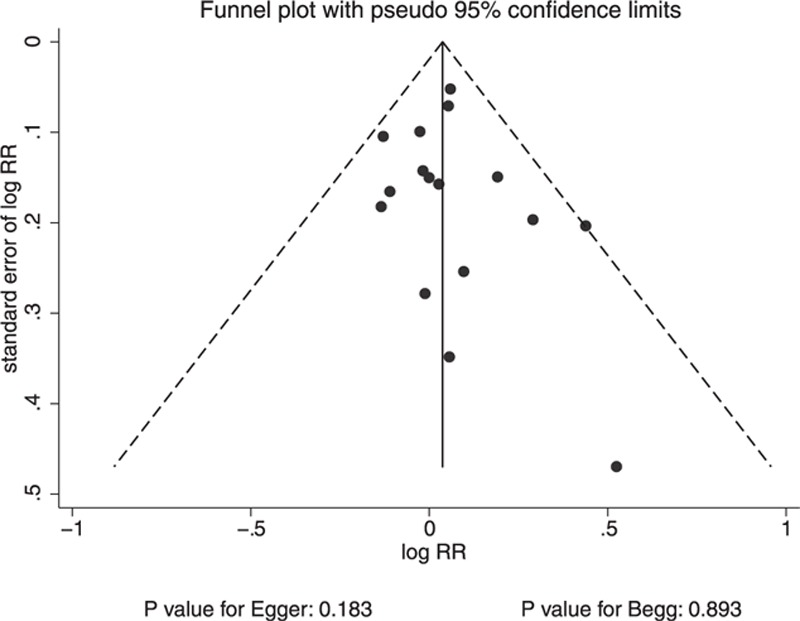
Funnel plot for cancer incidence.

A review of funnel plots did not rule out the potential for publication bias for cancer incidence. The Egger^[37]^ and Begg tests,^[38]^ however, showed no evidence of publication bias for cancer incidence (*P* value for Egger: 0.183; *P* value for Begg: 0.893; Fig. 5).

## 4 Discussion

Previous observational studies^[39–46]^ have suggested that vitamin B supplementation has a marked effect on cancer incidence. However, observational studies may overestimate the effect of vitamin B supplementation. So far, the effect of vitamin B supplementation on the risk of cancer has not been confirmed by any RCT. We therefore conducted a meta-analysis of RCTs to quantitatively assess the effect of vitamin B supplementation on the risk of cancer-related outcomes, and the findings of our study, which has a large sample size, are potentially more robust than those of any individual trial. In this study, RCTs were included to explore all possible correlations between vitamin B supplementation and the outcomes of cancer incidence, death due to cancer, and total mortality. This comprehensive, large-scale, quantitative study included 74,498 individuals from 18 trials with a broad range of baseline characteristics. The findings of our study indicated that vitamin B supplementation has no significant effect on cancer incidence, death due to cancer, and total mortality. Considering specific cancer types, vitamin B significantly reduced the risk of skin melanoma, but did not have any significant effect on other types of cancers. In a cumulative meta-analysis, the effect of vitamin B on cancer incidence, death due to cancer, and total mortality persisted and remained nonsignificant.

Several meta-analyses have evaluated the impact of vitamin B supplementation on the risk of cancer-related outcomes.^[26,27,47–54]^ For meta-analysis based on observational studies, folic acid supplementation was associated with a lower risk of oral and pharyngeal,^[47]^ breast,^[48]^ bladder,^[49]^ esophageal and pancreatic cancer.^[50]^ Furthermore, dietary folic acid supplementation was not associated with the risk of colorectal,^[51]^ prostate,^[52]^ lung,^[53]^ and gastric cancer.^[50]^ In addition, Zhang et al^[53]^ suggested that folic acid supplementation might affect subsequent lung cancer risk in men. Tio et al^[52]^ indicated that high blood folate level was associated with an increased risk of prostate cancer. However, the hypothesized effect of vitamin B supplementation comes from meta-analyses of observational studies, which may overestimate its effect on the incidence of specific types of cancer. Three important systematic reviews and meta-analyses of RCTs have evaluated the impact of folic acid supplementation on cancer incidence and have found no evidence to support a significant effect.^[26,27,54]^ Clarke et al^[26]^ performed a meta-analysis of 8 RCTs involving 37,485 individuals and found that vitamin B supplementation had no significant effect on cancer incidence (RR, 1.05; 95% CI: 0.98–1.13), cancer mortality (RR, 1.00; 95% CI: 0.85–1.18), and total mortality (RR, 1.02; 95% CI: 0.97–1.08) during the whole scheduled treatment period or in the subsequent years. Vollset et al^[27]^ suggested that folic acid supplementation was associated with higher plasma concentrations of folic acid, but had no significant effect on cancer incidence. For specific types of cancers, there was no significant difference between vitamin B and placebo for cancer at any specific sites. Qin et al^[54]^ indicated that while folic acid supplementation had no significant effect on total cancer incidence, and the incidence of colorectal, prostate, lung, breast, and hematological malignancy cancers, it significantly reduced the risk of melanoma. In the present study, all pooled RR estimate points for cancer incidence were >1 (evidence accumulated up to 2006) with a potential trend toward moving rightward in the cumulative meta-analysis of vitamin B supplementation. A potentially harmful effect of vitamin B on total cancer incidence was detected, but this trend was nonsignificant and requires validation. For death due to cancer and total mortality, the nonsignificant effects persisted and remained.

There was no significant difference between vitamin B supplementation and placebo in terms of the effect on the relative risks of cancer incidence, death due to cancer, and total mortality. Cumulative findings of out meta-analysis indicated, with evidence accumulated up to 2006, that the pooled RR estimate points for cancer incidence were >1. A study conducted by Ulrich and Potter^[55]^ indicated that folic acid may have influenced growth in cancers that were silent at baseline or during trials, leading to excess subsequent clinical surfacing and diagnosis during extended follow-up. Furthermore, evidence suggests that aggressive supplementation may enhance the growth of established, microscopic lesions.^[56]^ Data on the incidence of specific types of cancer were available in our study; however, no significant difference was detected between vitamin B supplementation and placebo, except in the case of skin melanoma. These results may be attributed to chance, because a small number of trials^[10,16,20]^ were included.

Subgroup analyses were conducted for cancer incidence, death due to cancer, and total mortality. No significant effect on cancer incidence was observed in subpopulations with a mean age less than 62 years and those with a baseline homocysteine level >14 μmol/L; the effect seemed to be slight, but nonsignificant. In the current study, mean age and baseline homocysteine levels in participants were available for whole populations; individual data were not available, which prevented us from performing more comprehensive analyses. Furthermore, participants with different backgrounds and intervention regimens might contribute to the biased treatment effect. Finally, nearly all included trials included participants from the Western countries except 1 trial, which specifically included Chinese people.^[24]^ The findings of CSPPT were consistent with those of trials conducted in Western countries. Furthermore, alimentation habits might play an important role in the risk of cancer;^[24]^ however, data about alimentation status were not available to us. Therefore, we just performed a relative comprehensive review to evaluate the effect of vitamin B on the risk of cancer, death due to cancer, and total mortality.

The present meta-analysis has certain limitations. First, different types and doses of vitamin B supplements were included, which could bias the results. Second, the background among participants taking vitamin B might have impaired our ability to identify the treatment effect. Third, the different results of cancer surveillance and reporting may lead to various incidences of cancer among trials. Fourth, patients who have had background therapy for previous diseases were not available in stratified analyses. Fifth, several included trials with low Jadad score, which hampered the quality of our work. Finally, more detailed relevant analysis could be restricted by conducting analysis using pooled data instead of individual data.

In conclusion, vitamin B supplementation has no significant effect on cancer incidence, death due to cancer, and total mortality. Subgroup analyses suggested that vitamin B might have a detrimental effect on cancer incidence when the mean age of the participants was less than 62 years and baseline homocysteine levels were >14 μmol/L. In addition, vitamin B supplementation significantly reduced the risk of skin melanoma. Future trials should focus on specific younger subpopulations and participants with baseline homocysteine level >14 μmol/L. We suggest that ongoing trials should be improved in the following ways: total cancer incidence, and death due to cancer or any specific type of cancer should be recorded and reported normatively, and it should be evaluated in future trials, and the role of intervention duration and dosage of supplementation should be taken into consideration before evaluating clinical outcomes.

## References

[1] Larsson SC Giovannucci E Wolk A Folate and risk of breast cancer: a meta-analysis. *J Natl Cancer Inst* 2007; 99:64–76. 17202114 10.1093/jnci/djk006

[2] Giovannucci E Epidemiologic studies of folate and colorectal neoplasia: a review. *J Nutr* 2002; 132 (suppl):2350S–2355S. 12163691 10.1093/jn/132.8.2350S

[3] Sanjoaquin MA Allen NA Couto E Folate intake and colorectal cancer risk: a meta-analytical approach. *Int J Cancer* 2005; 113:825–838. 15499620 10.1002/ijc.20648

[4] Smith AD Kim YI Refsum H Is folic acid good for everyone? *Am J Clin Nutr* 2008; 87:517–533. 18326588 10.1093/ajcn/87.3.517

[5] Liu L Wylie RC Andrews LG Aging, cancer and nutrition: the DNA methylation connection. *Mech Ageing Dev* 2003; 124:989–998. 14659588 10.1016/j.mad.2003.08.001

[6] Ebbing M B⊘naa KH Nygard O Cancer incidence and mortality after treatment with folic acid and vitamin B12. *JAMA* 2009; 302:2119–2126. 19920236 10.1001/jama.2009.1622

[7] Zhou Y-H Tang J-Y Wu M-J Effect of folic acid supplementation on cardiovascular outcomes: a systematic review and meta-analysis. *PLoS One* 2011; 6:e25142. 21980387 10.1371/journal.pone.0025142 PMC3182189

[8] Heinz J Kropf S Domröse U B vitamins and the risk of total mortality and cardiovascular disease in end-stage renal disease: results of a randomized controlled trial. *Circulation* 2010; 121:1432–1438. 20231532 10.1161/CIRCULATIONAHA.109.904672

[9] The VISP Trial Investigators. Lowering homocysteine in patients with ischemic stroke to prevent recurrent stroke, myocardial infarction, and death: the vitamin intervention for stroke prevention (VISP) randomized controlled trial. *JAMA* 2004; 291:565–575. 14762035 10.1001/jama.291.5.565

[10] The Heart Outcomes Prevention Evaluation (HOPE) 2 Investigators. Homocysteine lowering with folic acid and B vitamins in vascular disease. *N Engl J Med* 2006; 354:1567–1577. 16531613 10.1056/NEJMoa060900

[11] The NORVIT Trial Investigators. Homocysteine lowering and cardiovascular events after acute myocardial infarction. *N Engl J Med* 2006; 354:1578–1588. 16531614 10.1056/NEJMoa055227

[12] Righetti M Serbelloni P Milani S Homocysteine-lowering vitamin B treatment decreases cardiovascular events in hemodialysis patients. *Blood Purif* 2006; 24:379–386. 16755160 10.1159/000093680

[13] The Polyp Prevention Study Group. Folic acid for the prevention of colorectal adenomas a randomized clinical trial. *JAMA* 2007; 297:2351–2359. 17551129 10.1001/jama.297.21.2351

[14] The Veterans Affairs Site Investigators. Effect of homocysteine lowering on mortality and vascular disease in advanced chronic kidney disease and end-stage renal disease: a randomized controlled trial. *JAMA* 2007; 298:1163–1170. 17848650 10.1001/jama.298.10.1163

[15] The WENBIT Study Group. Mortality and cardiovascular events in patients treated with homocysteine-lowering B vitamins after coronary angiography: a randomized controlled trial. *JAMA* 2008; 300:795–804. 18714059 10.1001/jama.300.7.795

[16] The WAFACS Study Group. Effect of combined folic acid, vitamin B6, and vitamin B12 on cancer risk in women: a randomized trial. *JAMA* 2008; 300:2012–2021. 18984888 10.1001/jama.2008.555 PMC2593624

[17] The BVAIT Research Group. High-dose B vitamin supplementation and progression of subclinical atherosclerosis: a randomized controlled trial. *Stroke* 2009; 40:730–736. 19118243 10.1161/STROKEAHA.108.526798 PMC2701290

[18] Study of the Effectiveness of Additional Reductions in Cholesterol and Homocysteine (SEARCH) Collaborative Group. Effects of homocysteine-lowering with folic acid plus vitamin B12 vs placebo on mortality and major morbidity in myocardial infarction survivors: a randomized trial. *JAMA* 2010; 303:2486–2494. 20571015 10.1001/jama.2010.840

[19] The SU.FOL.OM3 Collaborative Group. Effects of B vitamins and omega 3 fatty acids on cardiovascular diseases: a randomised placebo controlled Trial. *BMJ* 2010; 341:c6273. 21115589 10.1136/bmj.c6273 PMC2993045

[20] The VITAmins TO Prevent Stroke (VITATOPS) Trial Study Group. Treatment with B vitamins and incidence of cancer in patients with previous stroke or transient ischemic attack results of a randomized placebo-controlled trial. *Stroke* 2012; 43:1572–1577. 22474057 10.1161/STROKEAHA.111.641613

[21] The ukCAP Trial Group. Aspirin and folic acid for the prevention of recurrent colorectal adenomas. *Gastroenterology* 2008; 134:29–38. 18022173 10.1053/j.gastro.2007.10.014

[22] The Vitamins and Thrombosis (VITRO) Study Group. Homocysteine lowering by B vitamins and the secondary prevention of deep vein thrombosis and pulmonary embolism: a randomized, placebo-controlled, double-blind trial. *Blood* 2007; 109:139–144. 16960155 10.1182/blood-2006-04-014654

[23] Wu K Platz EA Willett WC A randomized trial on folic acid supplementation and risk of recurrent colorectal adenoma. *Am J Clin Nutr* 2009; 90:1623–1631. 19864409 10.3945/ajcn.2009.28319 PMC2777472

[24] CSPPT Investigators. Efficacy of folic acid therapy in primary prevention of stroke among adults with hypertension in China: the CSPPT randomized clinical trial. *JAMA* 2015; 313:1325–1335. 25771069 10.1001/jama.2015.2274

[25] B-PROOF Study Group. Effect of daily vitamin B-12 and folic acid supplementation on fracture incidence in elderly individuals with an elevated plasma homocysteine concentration: B-PROOF, a randomized controlled trial. *Am J Clin Nutr* 2014; 100:1578–1586. 25411293 10.3945/ajcn.114.090043

[26] Clarke R Halsey J Lewington S Effects of lowering homocysteine levels with B vitamins on cardiovascular disease, cancer, and cause-specific mortality: meta-analysis of 8 randomized trials involving 37 485 individuals. *Arch Intern Med* 2010; 170:1622–1631. 20937919 10.1001/archinternmed.2010.348

[27] Vollset SE Clarke R Lewington S Effects of folic acid supplementation on overall and site-specific cancer incidence during the randomised trials: meta-analyses of data on 50,000 individuals. *Lancet* 2013; 381:1029–1036. 23352552 10.1016/S0140-6736(12)62001-7 PMC3836669

[28] Moher D Liberati A Tetzlaff J PRISMA Group. Preferred reporting items for systematic reviews and meta-analyses: the PRISMA statement. *PLos Med* 2009; 6:e1000097. 19621072 10.1371/journal.pmed.1000097 PMC2707599

[29] Jadad AR Moore RA Carroll D Assessing the quality of reports of randomized clinical trials: is blinding necessary? *Control Clin Trials* 1996; 17:1–12. 8721797 10.1016/0197-2456(95)00134-4

[30] DerSimonian R Laird N Meta-analysis in clinical trials. *Control Clin Trials* 1986; 7:177–188. 3802833 10.1016/0197-2456(86)90046-2

[31] Ades AE Lu G Higgins JP The interpretation of random-effects metaanalysis in decision models. *Med Decis Making* 2005; 25:646–654. 16282215 10.1177/0272989X05282643

[32] Deeks JJ Higgins JPT Altman DG Higgins J Green S Analyzing data and undertaking meta-analyses. *Cochrane Handbook for Systematic Reviews of Interventions 5. 0. 1*. Oxford, UK: The Cochrane Collaboration; 2008; chap 9.

[33] Higgins JPT Thompson SG Deeks JJ Measuring inconsistency in meta-analyses. *BMJ* 2003; 327:557–560. 12958120 10.1136/bmj.327.7414.557 PMC192859

[34] Thompson SG Higgins JP How should meta-regression analyses be undertaken and interpreted? *Stat Med* 2002; 21:1559–1573. 12111920 10.1002/sim.1187

[35] Altman DG Bland JM Interaction revisited: the difference between two estimates. *BMJ* 2003; 326:219. 12543843 10.1136/bmj.326.7382.219 PMC1125071

[36] Tobias A Assessing the influence of a single study in meta-analysis. *Stata Tech Bull* 1999; 47:15–17.

[37] Egger M Davey Smith G Schneider M Bias in meta-analysis detected by a simple, graphical test. *BMJ* 1997; 315:629–634. 9310563 10.1136/bmj.315.7109.629 PMC2127453

[38] Begg CB Mazumdar M Operating characteristics of a rank correlation test for publication bias. *Biometrics* 1994; 50:1088–1101. 7786990

[39] Su LJ Arab L Nutritional status of folate and colon cancer risk: evidence from NHANES I epidemiologic follow-up study. *Ann Epidemiol* 2001; 11:65–72. 11164122 10.1016/s1047-2797(00)00188-5

[40] Wei EK Giovannucci E Wu K Comparison of risk factors for colon and rectal cancer. *Int J Cancer* 2004; 108:433–442. 14648711 10.1002/ijc.11540 PMC2903217

[41] Ferraroni M La Vecchia C D’Avanzo B Selected micronutrient intake and the risk of colorectal cancer. *Br J Cancer* 1994; 70:1150–1155. 7981067 10.1038/bjc.1994.463 PMC2033709

[42] Lajous M Romieu I Sabia S Folate, vitamin B12 and postmenopausal breast cancer in a prospective study of French women. *Cancer Causes Control* 2006; 17:1209–1213. 17006726 10.1007/s10552-006-0053-3 PMC1925055

[43] Levi F Pasche C Lucchini F Dietary intake of selected micronutrients and breast-cancer risk. *Int J Cancer* 2001; 91:260–263. 11146455 10.1002/1097-0215(200002)9999:9999<::aid-ijc1041>3.3.co;2-r

[44] Shrubsole MJ Jin F Dai Q Dietary folate intake and breast cancer risk: results from the Shanghai Breast Cancer Study. *Cancer Res* 2001; 61:7136–7141. 11585746

[45] Adzersen KH Jess P Freivogel KW Raw and cooked vegetables, fruits, selected micronutrients, and breast cancer risk: a case-control study in Germany. *Nutr Cancer* 2003; 46:131–137. 14690788 10.1207/S15327914NC4602_05

[46] Lajous M Lazcano-Ponce E Hernandez-Avila M Folate, vitamin B(6), and vitamin B(12) intake and the risk of breast cancer among Mexican women. *Cancer Epidemiol Biomarkers Prev* 2006; 15:443–448. 16537699 10.1158/1055-9965.EPI-05-0532

[47] Galeone C Edefonti V Parpinel M Folate intake and the risk of oral cavity and pharyngeal cancer: a pooled analysis within the International Head and Neck Cancer Epidemiology Consortium. *Int J Cancer* 2015; 136:904–914. 24974959 10.1002/ijc.29044 PMC4262536

[48] Chen P Li C Li X Higher dietary folate intake reduces the breast cancer risk: a systematic review and meta-analysis. *Br J Cancer* 2014; 110:2327–2338. 24667649 10.1038/bjc.2014.155 PMC4007237

[49] He H Shui B Folate intake and risk of bladder cancer: a meta-analysis of epidemiological studies. *Int J Food Sci Nutr* 2014; 65:286–292. 24328495 10.3109/09637486.2013.866641

[50] Tio M Andrici J Cox MR Folate intake and the risk of upper gastrointestinal cancers: a systematic review and meta-analysis. *J Gastroenterol Hepatol* 2014; 29:250–258. 24224911 10.1111/jgh.12446

[51] Heine-Bröring RC Winkels RM Renkema JM Dietary supplement use and colorectal cancer risk: a systematic review and meta-analyses of prospective cohort studies. *Int J Cancer* 2015; 136:2388–2401. 25335850 10.1002/ijc.29277

[52] Tio M Andrici J Cox MR Folate intake and the risk of prostate cancer: a systematic review and meta-analysis. *Prostate Cancer Prostatic Dis* 2014; 17:213–219. 24819234 10.1038/pcan.2014.16

[53] Zhang YF Zhou L Zhang HW Association between folate intake and the risk of lung cancer: a dose-response meta-analysis of prospective studies. *PLoS One* 2014; 9:e93465. 24713625 10.1371/journal.pone.0093465 PMC3979671

[54] Qin X Cui Y Shen L Folic acid supplementation and cancer risk: a meta-analysis of randomized controlled trials. *Int J Cancer* 2013; 133:1033–1041. 23338728 10.1002/ijc.28038

[55] Ulrich CM Potter JD Folate and cancer: timing is everything. *JAMA* 2007; 297:2408–2409. 17551134 10.1001/jama.297.21.2408

[56] im Y-I Salomon RN Graeme-Cook F Dietary folate protects against the development of macroscopic colonic neoplasia in a dose responsive manner in rats. *Gut* 1996; 39:732–740. 9014775 10.1136/gut.39.5.732 PMC1383400

